# The Therapeutic Effect of Ge-Gen Decoction on a Rat Model of Primary Dysmenorrhea: Label-Free Quantitative Proteomics and Bioinformatic Analyses

**DOI:** 10.1155/2020/5840967

**Published:** 2020-12-01

**Authors:** Yazhen Xie, Jianqiang Qian, Qibin Lu

**Affiliations:** ^1^Taicang Traditional Chinese Medicine Hospital, Affiliated to Nanjing University of Chinese Medicine, Taicang 215400, China; ^2^Department of Gynaecology, The Affiliated Hospital of Nanjing University of Chinese Medicine, Nanjing 210029, China

## Abstract

Ge-Gen decoction (GGD) is widely used for the treatment of primary dysmenorrhea (PD) in China. However, the mechanisms that underlie this effect are unclear. We investigated the protective mechanism of GGD in a rat model of PD using label-free quantitative proteomics. The model was established by the administration of estradiol benzoate and oxytocin. Thirty rats were divided into three groups (ten rats/group): a control group (normal rats), a model group (PD rats), and a treatment group (PD rats treated with GGD). The serum levels of prostaglandin E2 (PGE2) and pros*taglandi*n F2*α* (PGF2*α*) were measured by ELISA. Nanohigh-performance liquid chromatography-tandem mass spectrometry (nano-HPLC-MS/MS) was used to identify differentially expressed proteins (DEPs), and bioinformatics was used to investigate the protein function. Proteomic data were validated by western blot analysis. Oxytocin-induced writhing responses and abnormal serum levels of PGE2 and PGF2*α* were reversed following the administration of GGD. A total of 379 DEPs were identified; 276 were identified between the control group and the model group, 144 were identified between the model group and the treatment group, and 41 were identified as DEPs that were common to all groups. Bioinformatics revealed that the DEPs between the control group and the model group were mainly associated with cellular component biogenesis and binding processes. The DEPs between the model group and the treatment group were mainly involved in the protein binding and metabolic process. The expression levels of HSP90AB1 and the phosphorylation levels of ERK, JNK, and P-p38 in the uteri of rats in the three groups were consistent with the proteomic findings; MAP kinases (ERK, JNK, and p38) are known to be involved in the production of inflammatory cytokines and oxytocin signaling while HSP90AB1 is known to be associated with estrogen signaling. Collectively, these data indicate that GGD may exert its protective function on PD by regulating the inflammatory response and signaling pathways associated with oxytocin and estrogen.

## 1. Introduction

Primary dysmenorrhea (PD) refers to recurrent menstrual cramps caused by uterine contractions [1, 2]. This condition affects 40%–50% of adolescent women and can have a disruptive effect on a patient's quality of life [1–3]. The most common medications for treating PD are oral contraceptives, analgesics, and nonsteroidal anti-inflammatory drugs (NSAIDs) [4, 5]. However, these drugs may induce a range of side effects and are associated with a failure rate of up to 25% [4, 5]. Thus, there is an urgent need to develop new, safe, and effective, therapeutic options for the treatment of PD.

Herbal medicine has been used in China for many years to treat PD [6–10]. Ge-Gen decoction (GGD, Kakkon-to in Japanese) is a traditional Chinese prescription that is widely applied in the clinical treatment of PD [11, 12]. GGD is developed from *Shang Han Za Bing Lun* and is composed of *Pueraria lobata* (Ge Gen), *Ephedrae* (Ma Huang), *cinnamon twig* (Gui Zhi), *Paeonia lactiflora* (Shao Yao), *ginge*r (Sheng Jiang), *Glycyrrhizae radix et rhizome* (Zhi Gan Cao), and *red dates* (Hong Zao) in a specific ratio (4 : 1 : 3 : 3 : 2 : 2 : 4) [13, 14]. Previous studies revealed that *Paeonia lactiflora*, *Glycyrrhizae Radix et Rhizoma*, and *ging*er could exert antidysmenorrhea effects [12]. Cinnamic acid and cinnamaldehyde, the two major constituents of *cinnamon twig*, have also been reported to inhibit uterine contractions induced by oxytocin [10, 15]. Furthermore, ephedrine, a major constituent of *Ephedrae*, has been shown to relax smooth muscle and activate *β*-adrenoceptors [12, 14]. However, the molecular mechanisms that underlie the protective effect of GGD in patients with PD have yet to be elucidated.

In this study, we used label-free quantitative proteomics to investigate the effects of the GGD administration in a rat model of PD.

## 2. Materials and Methods

### 2.1. GGD Preparation

GGD was obtained by the Jiangsu Province Hospital of Chinese Medicine (Jiangsu, China) and consisted of seven components (Table 1). The principal components of GGD were prepared and systematically identified using methodology described previously [14].

### 2.2. Animal Model Establishment

Thirty clean, healthy, mature, and unmated Wistar female rats (weighing 200 ± 20 g; 8–10 weeks-of-age) were obtained from Cavens Laboratory Animal Co., Ltd. (Changzhou, China). Next, we used established methods to create painful models of menstruation (cold-damp congealing and stagnation types) [16]. Rats were maintained at a room temperature of 23 ± 2°C. Once a day, the posterior limbs and hypogastrium of each rat were immersed into ice-water mixture (0 ± 1°C) for 20 mins at a time; this created a cold stimulus. In addition, the head of each rat was injected once a day (subcutaneously) with estradiol benzoate for 10 consecutive days; on days 1 and 10, the dose was 0.5 mg/rat, while on days 2–9, the dose was reduced to 0.2 mg/rat. The rats experiencing these treatments experienced a range of symptoms, including shivering, hollowed back and piloerection, sneezing, cowering and hypokinesia, low spirits, loose stools, reduced food intake, and reduced drinking; these animals also had pale mouths, lips, ears, noses, claws, nails, and tails. These manifestations relate directly to the symptoms of cold-damp congealing and stagnation, thus indicating that the models had been established successfully. On the 11th day of modeling, each rat was given an intraperitoneal injection of oxytocin (2 U/rat). Rats in the blank group received subcutaneous injections of normal saline (at an equivalent dose) in their heads. The models of painful menstruation were established successfully, as evidenced by contractions and indentations on the abdomen, trunk, and posterior of each of the experimental rats. Seventy-five min after modeling on the 8th day, each rat was given GGD by the intragastric administration at a dose of 2 mL/rat (the dose was converted based on the superficial area ratio between rats and humans: 0.018 × 20 g/0.2 kg = 1.8 g/kg; this was prepared with distilled water at a concentration of 18 g/100 mL). The treatment was conducted once a day for three consecutive days. All experiments were approved by the Ethics Committee of Taicang Hospital of traditional Chinese Medicine.

### 2.3. Writhing Test

Writhing scores were used to evaluate the writhing reaction of rats in response to pain, as described previously [16–18]. The writhing times of each rat were observed within 30 mins of the injection of oxytocin. Writhing scores were divided into four grades: 0–3 0, normal posture (foot pawns box or normal probing behavior); 1, body oblique side; 2, hind limb extension, hind paw dorsiflexion, body extension with frequent pelvic lateral rotation; and 3, abdominal muscle contraction and hind limb extension. Writhing scores were calculated according to the following formula: grade 0 × 0 + grade 1 × 1 + grade 2 × 2 + grade 3 × 3.

### 2.4. Enzyme-Linked Immunosorbent Assay (ELISA)

Blood samples were collected from the retroorbital plexus of each rat following the administration of GGD. Serum levels of PGE2, PGF2*α*, TNF-*α*, and IL-8 were then measured with specific ELISA kits, as described in the manufacturer's guidelines.

### 2.5. Proteomics

#### 2.5.1. Sample Preparation and Protein Digestion

The uteri of three rats per group were collected. The uterine tissue was first cut into smaller pieces and mixed with radioimmunoprecipitation assay (RIPA) buffer prior to three rounds of mechanical homogenization on ice (3 s per round). Samples were then centrifuged, and the supernatants were separately transferred into new microcentrifuge tubes for analysis. The bicinchoninic acid (BCA) assay was then used to determine the protein concentration in each supernatant. Proteins were then diluted with urea solution (8 M) followed by further incubation for 1 h at 37°C. Thereafter, the mixture was transferred into 10 K Microcon centrifugal filter units (Millipore, Billerica, MA, USA) and centrifuged to remove urea. Finally, the proteins were alkylated by iodoacetamide (55 mM) for 20 min at room temperature (in the dark), digested with sequence-grade modified trypsin (Promega, Madison, WI, USA), and lyophilized.

#### 2.5.2. Liquid Chromatographymass Spectrometry/Mass Spectrometry (LC-MS/MS) Analysis

Peptides were treated with formic acid (0.1%, 30 *μ*L), loaded into a trap column (C18, 75 *μ*m × 2 cm, flow rate: 300 nL/min), and subsequently separated and loaded onto an analytical column (C18, 75 *μ*m × 50 cm) using a linear gradient of 5–38% formic acid (0.1%) for 120 min. Throughout the study, we used an electrospray voltage of 2 kV between the sprayer and the ion inlet of the mass spectrometer.

#### 2.5.3. Identification of Differentially Expressed Proteins (DEPs)

MaxQuant software (https://www.maxquant.org/)was used to analyze the tandem mass spectra in relation to the UniProtKB database for *Rattus norvegicus*. The search parameters for the mass tolerances of the fragment and parent ions were 0.05 and 7 ppm, respectively. The fixed modification was carbamidomethylation, while the variable modifications were deamidation (NQ), oxidation (M), and acetylation (Protein N-term). Peptides were filtered with a 1% false discovery rate (FDR) and one unique. The relative abundance of peptides and proteins were compared using analysis of variance (ANOVA). The mean abundance of all peptides was normalized by the application of medians. A protein with a fold change > 1.5, and the presence of least 2 unique peptides with a *P* value <0.05, was considered to be differentially expressed protein (DEP).

#### 2.5.4. Bioinformatic Analysis of DEPs

Once identified, DEPs were then analyzed by reference to three key databases: Kyoto Encyclopedia of Genes and Genomes (KEGG, http://kobas.cbi.pku.edu.cn), Gene Ontology (GO, http://www.geneontology.org/), and the Clusters of Orthologous Groups (KOGs, http://www.ncbi.nlm.nih.gov/COG/). We also used STRING (http://string-db.org/) to create an interaction network for the DEPs.

### 2.6. Western Blotting

Proteins from the uteri of rats in each treatment group were extracted using RIPA lysis buffer (Beyotime, Haimen, China). We then used the BCA assay to estimate the concentration of each protein extract. Proteins were then separated by 8–10% sodium dodecyl sulfate-polyacrylamide gel electrophoresis (SDS-PAGE) and transferred to polyvinylidene difluoride (PVDF) membranes. After blocking with nonfat milk (5%), and the PVDF membranes were incubated overnight at 4°C with the following antibodies: rabbit anti-P-JNK (Cell Signaling Technology, 4668, dilution:1/1000), rabbit anti-JNK (Cell Signaling Technology, 9252, dilution:1/1000), rabbit anti-P-ERK (Cell Signaling Technology, 4370, dilution:1/2000), rabbit anti-ERK (Cell Signaling Technology, 5013, dilution:1/1000), rabbit anti-P-P38 (Cell Signaling Technology, 4511, dilution:1/1000), rabbit anti-P38 (Cell Signaling Technology, 8690, dilution:1/2000), rabbit anti-HSP90AA (Cell Signaling Technology, 8165, dilution:1/1000), rabbit anti-HSP90AB (Cell Signaling Technology, 5087, dilution:1/1000), and anti-*β*-actin (Cell Signaling Technology, 5174, dilution:1/1000). *β*-Actin was used as a loading control. After washing three times with Tris Buffered saline Tween (TBST) buffer, the membranes were incubated for 1 h at room temperature with goat anti-rabbit antibody (Santa Cruz, sc-2004, dilution:1/5000). Enhanced chemiluminescence (ECL) reagent (Thermo Fisher Scientific Inc.) was then used to detect protein bands showing positive immunoreactivity. ImageJ software (version 1.48, National Institutes of Health, USA) was used to compare the relative optical densities of each band of interest.

### 2.7. Statistical Analysis

SPSS version 19.0 software (IBM Corp., NY) was used for all statistical analyses. Differences between the two groups were analyzed by Student's *t*-test. One-way ANOVA was used to make comparisons between multiple groups. Results are expressed as the mean ± standard deviation. Statistical significance was assumed at *P* < 0.05.

## 3. Results

### 3.1. GGD Attenuated the Oxytocin-Induced Writhing Response and Reduced the Serum Levels of PGE2 and PGF2*α* in a Rat Model of PD

**Figure 1:**
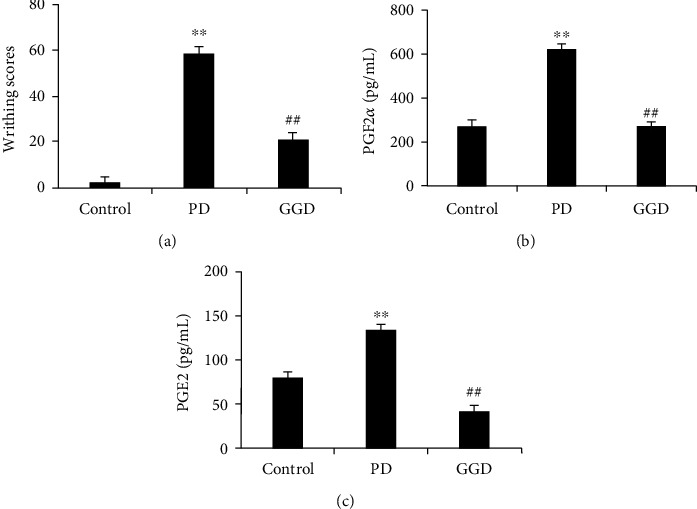
Effects of Ge-Gen decoction on writhing scores and the serum levels of PGF2*α* and PGE2 in a rat model of PD. Data represent mean ± SD (*n* = 10); ^**^P < 0.01 versus normal control group; ##*P* < 0.01 versus PD group.

The writhing scores of rats in the model group were significantly higher than those in the control group (*P* < 0.05; Figure 1(a)), thus suggesting that the model had been established successfully. Treatment with GGD led to a remarkable reduction in writhing scores (*P* < 0.05, Figure 1(a)).

We then measured the serum levels of PGE2 and PGF2*α* in each group of rats. As shown in Figure 1(b), the model group showed remarkable increases (*P* < 0.05) in the serum levels of PGE2 and PGF2*α*. However, the administration of GGD resulted in a remarkable reduction in the serum levels of PGE2 and PGF2*α* (*P* < 0.05). These findings suggested that GGD treatment could attenuate the oxytocin-induced writhing response.

### 3.2. Identification of DEPs in the Uterine Tissue

**Figure 2:**
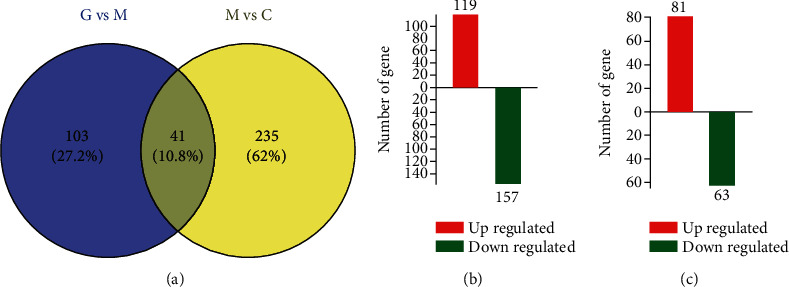
Differentially expressed proteins (DEPs) among the three groups. (a) Venn diagram showing overlapping DEPs. (b) DEPs between the model group and the treatment group. (c) DEPs between the model group and the control group. Upregulated proteins are indicated by red, while downregulated proteins are indicated by green. C represents the control group, M represents the model group, and G represents the treatment group.

Label-free quantitative proteomics was used to investigate the protective mechanisms of GGD. A total of 1968 protein groups, 4273 proteins, 10352 peptides, and 9311 unique peptides were successfully identified by LC-MS/MS among the three groups. The details of 379 DEPs are shown as Venn diagrams in Figure 2(a). A total of 276 DEPs were identified between the model group and the control group; of these, 157 were downregulated, while 119 were upregulated (Figure 2(b)). Moreover, a total of 144 DEPs were identified between the model group and the treatment group; 81 of these were upregulated, and 63 were downregulated (Figure 2(c)).

### 3.3. Bioinformatic Analysis of DEPs

**Figure 3:**
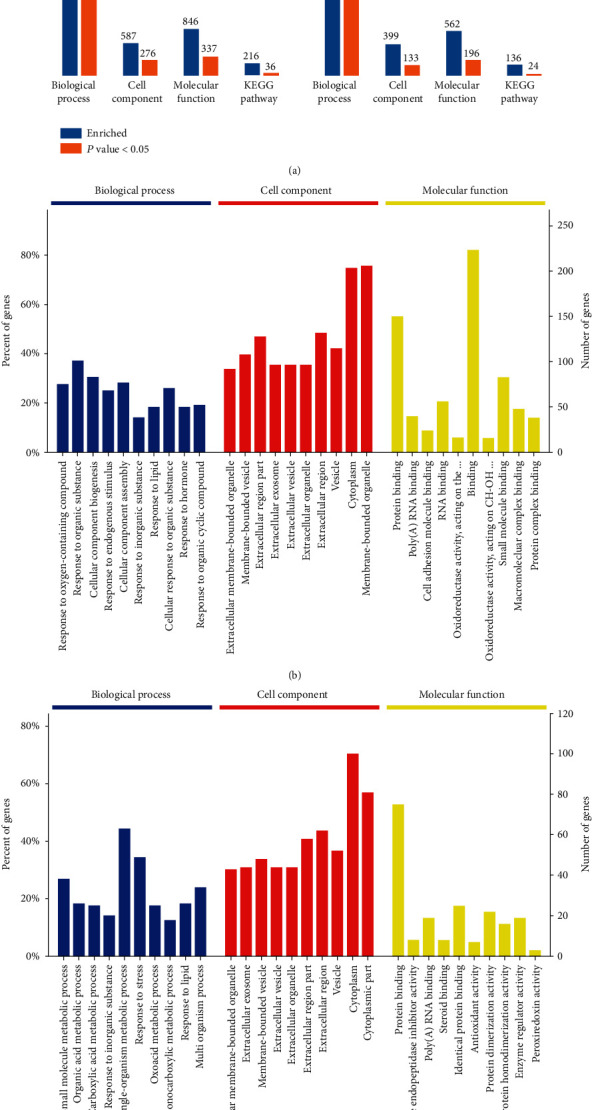
Functional annotation and categories of DEPs. (a) Bioinformatic analysis of the DEPs identified between the model group and the control group (left) and the DEPs identified between the model group and the treatment group (right) using BP, CC, MF, and KEGG pathway analysis. (b, c) The top ten significantly enriched GO terms for the DEPs identified between the model group and the control group (b) and the DEPs identified between the model group and the treatment group (c).

OmicsBean (http://www.omicsbean.cn/, Gene for health, Shanghai, China) was used to analyze the DEPs with regards to pathways and biological processes (BP). GO annotation of the DEPs between the model group and the control group identified a total of 276 cell component (CC) terms, 337 molecular function (MF) terms, and 1579 BP terms (Figure 3(a), left). Analysis of the DEPs between the model group and the treatment group identified 133 CC terms, 196 MF terms, and 977 BP terms (Figure 3(a), right). The top ten GO terms that showed remarkable enrichment (*P* < 0.05) are shown in Figures 3(b) and 3(c). BP analysis showed that the majority of the DEPs identified between the model group and the control group were classified as either ‘response to organic substance' and ‘cellular component biogenesis' (Figure 3(b)). With regards to CC terms, most of the DEPs identified between the model group and the control group were associated with the cytoplasm and membrane-bound organelles. With regards to the MF classification, the identified DEPs were predominantly involved in binding processes, and in particular, protein binding and small molecule binding. GO analysis of the DEPs identified between the model group and the treatment group showed that the greatest changes with regards to BP were associated with single-organism metabolic process and response to stress (Figure 3(c)). Most of the DEPs identified between the model group and the treatment group were cytoplasmic in origin, followed by the extracellular region. MF analysis further showed that these DEPs were most commonly associated with protein binding.

**Figure 4:**
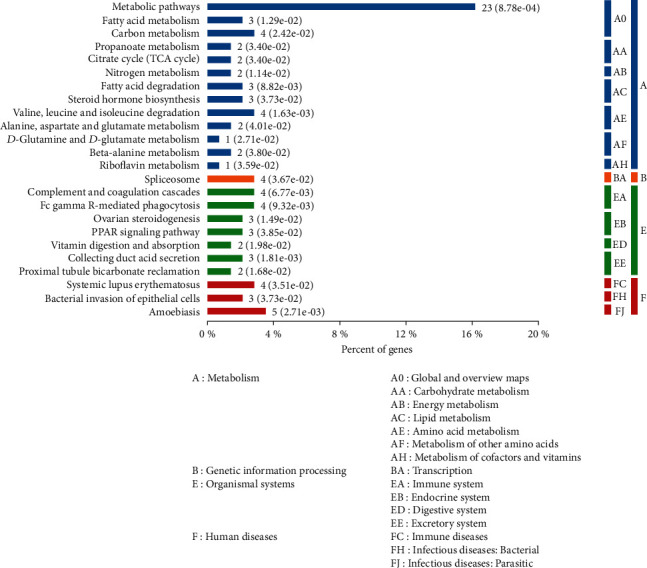
Distribution of the enriched KEGG pathways for the DEPs identified between the model group and the treatment group. The right side of the column shows the number of proteins involved in a specific pathway along with corresponding *P* values.

Next, the DEPs identified between the model group and the treatment group were analyzed with regards to the biological pathways that were involved in the protective effect of GGD. KEGG pathway annotation subsequently showed that the main signaling pathways undergoing modulation were those related to metabolism (Figure 4).

**Figure 5:**
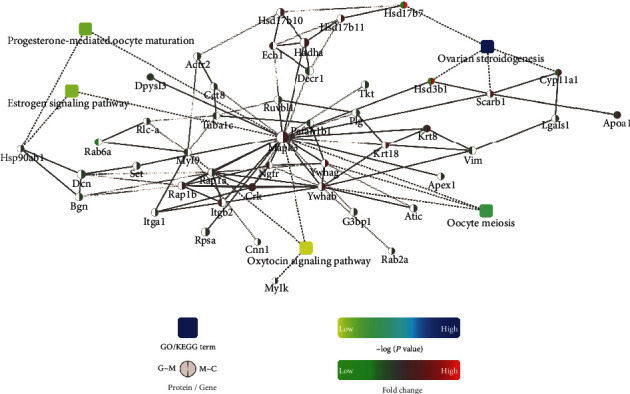
PPI analysis of DEPs. A protein association network was constructed for the DEPs according to the fold change of genes/proteins, KEGG pathway enrichment, protein-protein interaction, and biological process enrichment. Circle nodes with gradient colors (green, downregulation; red, upregulation) represent proteins. The DEPs identified between the model group and the treatment group are indicated by a left half of a circle, while the DEPs identified between the model group and the control group are indicated by a right half of a circle. Rectangles with a yellow to blue gradient color represent BPs. Yellow represents a smaller *P* value, while blue represents a larger *P* value.

To better understand the protective mechanisms of GGD, we used STRING to construct interaction networks for the DEPs among the three groups. These interactions revealed several BPs or signaling pathways that were related to the uterus, including oocyte meiosis, progesterone-mediated oocyte maturation, the oxytocin signaling pathway, ovarian steroidogenesis, and the estrogen signaling pathway. As shown in Figure 5, most of the DEPs featuring in the interaction map exhibited direct or indirect links. In particular, Mapk3 and Hsp90ab1 were involved in several different BPs or signaling pathways.

### 3.4. Validation of DEPs by Western Blotting

**Figure 6:**
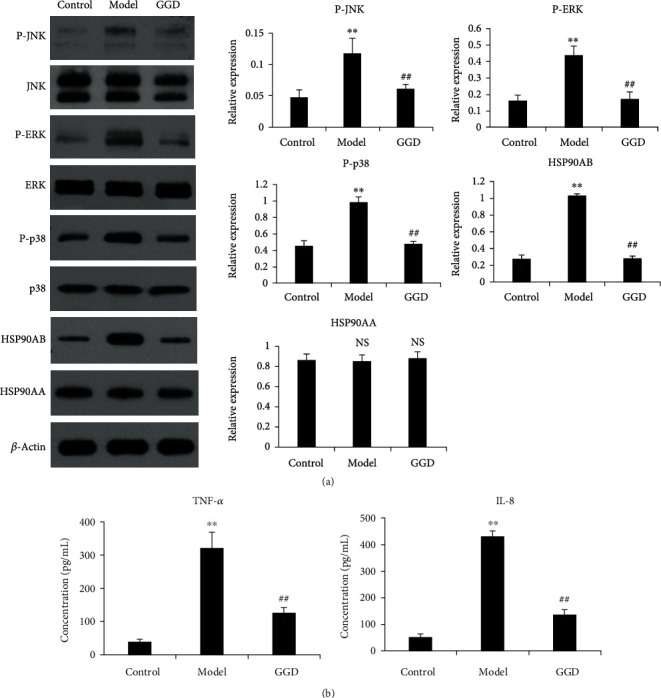
Western blotting validation of the DEPs identified by proteomics analysis. (a) Levels of P-JNK, P-ERK, P-p38, HSP90AA1 ,and HSP90AB1 in the uteri of rats in the three groups were determined by western blotting. (b) Serum levels of TNF-*α* and IL-8 in the three groups were measured by ELISA. ^∗∗^*P* < 0.01 versus control group; ##*P* < 0.01 versus model group. NS: no statistical significance.

As shown in Figure 6(a), the expression levels of HSP90AB1, and levels of phosphorylated ERK, JNK, and p38, were significantly increased in the model group (*P* < 0.05). However, the levels of these proteins were significantly reduced following the GGD administration. These findings were consistent with those derived from proteomic analysis. In addition, the serum levels of TNF-*α* and IL-8 were increased in the model group (*P* < 0.05, Figure 6(b)). Compared with the model group, the serum levels of TNF-*α* and IL-8 were significantly decreased in the treatment group (*P* < 0.05, Figure 6(b)).

## 4. Discussion

Previous clinical trials have demonstrated that GGD is an effective treatment for PD [12-14]. However, the mechanisms underlying these effects have yet to be elucidated. Mass spectrometry-based proteomics has been widely applied to investigate the mechanisms underlying the effects of traditional Chinese medicine (TCM) [19]. To the best of our knowledge, the present study is the first to use label-free quantitative proteomics to investigate the mechanisms of action responsible for the effect of GGD in a rat model of PD.

The main bioactive compounds of GGD were standardized previously via the application of high-performance liquid chromatography-quadrupole time-of-flight tandem mass spectrometry (HPLC-Q-TOF-MS/MS) [14]. A previous study showed that GGD inhibited uterine contractions in vitro and reduced writhing responses in a mouse model of PD [12]. Prostaglandins (PGs) are produced and released during menstruation, cause abnormal uterine contractions, and can also sensitize spinal neurons to pain [20–22]. PGE2 and PGF2*α*, two naturally occurring PGs, are regarded as the most crucial pain factors in PD [20–22]; these factors increase uterine contractility by binding to their respective receptors on the spiral arterioles [20–22]. Our analyses revealed that rats exposed to oxytocin experienced an elevation in writhing responses and increased levels of PGE2 and PGF2*α*; these effects were suppressed by GGD, thus suggesting that GGD may inhibit oxytocin-induced uterine contraction by regulating the levels of PGE2 and PGF2*α*. It is well known that PD is associated with increased levels of PGF2*α* [22]. However, the level of PGE2 in PD is controversial. PGE2 was reported to inhibit uterine contractions and relax the uterus [20–22]. However, PGE2 has also been shown to bind to receptors on the spiral arterioles, thus was shown to increase uterine contractility and lead to ischemic pain [23]. Increased levels of PGE2 in PD have also been reported in several models of PD [24, 25]. Therefore, we speculate that the level of PGE2 may be related to the type of the PD model used.

Our proteomic experiments identified a total of 379 DEPs. GO and KEGG analyses further revealed that the DEPs identified between the model group and the treatment group participated in various BPs and pathways, including cellular component biogenesis, protein binding, and metabolic pathways. These GO terms and pathways may play critical roles in the occurrence and development of PD. Several proteins were observed to recover after the administration of GGD. Protein-protein interaction (PPI) analysis further showed that these proteins were involved in signaling pathways related to oxytocin and estrogen and performed their functional roles collectively in specific networks.

Previous work has shown that inflammation is involved in PD, and that inflammatory cytokines are produced when inflammation occurs [26–29]. MAP kinases, including p38MAP kinase, c-Jun N-terminal kinase (JNK/SAPK), and extracellular signal-related kinase (ERK), play a critical role in the production of inflammatory cytokines [30–33]. Furthermore, these three kinases have also been shown to mediate oxytocin signaling [34, 35], which plays a critical role in the occurrence of PD. In the present study, the levels of P-ERK, P-JNK, and P-p38 were all significantly increased in rats with PD; however, the levels of these key factors all decreased following GGD treatment. Furthermore, it was evident that GGD treatment also significantly reduced the levels of inflammatory cytokines (TNF-*α* and IL-8). These results indicated that GGD may ameliorate oxytocin signaling and the inflammatory response in PD by reducing the activities of MAP kinases.

HSP90, a highly abundant molecular chaperone, has been shown to participate in the maintenance of proteostasis [36, 37]. HSP90 has also been shown to be involved in cellular adaptation to stress, the maintenance of homeostasis, and the functional maturation of steroid receptors [38]. The upregulation of HSP90 family members is closely associated with the progression of certain diseases, such as cancer, cystic fibrosis, and bronchopulmonary dysplasia [39–41]. There are two cytosolic isoforms of HSP90: HSP90AA1 and HSP90AB1 [38]. HSP90AA1 is known to localize in extracellular regions and plays an important role in tissue repair [42]. Other research has shown that HSP90AB1 plays a multitude of roles in a variety of human diseases by interacting with different proteins [43]. In the present study, we found that levels of HSP90AB1 increased significantly in PD rats but decreased following GGD treatment. This is important because estrogen has been shown to be essential for the progression of PD. In the present study, PPI analysis indicated that HSP90AB1 is involved in estrogen signaling. Therefore, it is reasonable to speculate that GGD could alleviate estrogen signaling in PD by reducing the expression of HSP90AB1.

The main active ingredients of GGD that show therapeutic effects against PD are considered to be *Pueraria lobata*, *Ephedra*, *Radix Paeoniae Alba*, and *Ramulus Cinnamomi*. Previous studies have reported that puerarin exerts an inhibitory effect on the production of NO, PGE2, and proinflammatory cytokines [44, 45]. *Ephedra* is also known to exert analgesic effects; the ephedrine and pseudoephedrine contained in *Ephedra* can activate the p38 MAPK pathway and inhibit the release of proinflammatory factors [46–48]. In a previous study, Kobayashi et al. [49] found that *Radix Paeoniae Alba* exerted an analgesic effect in an animal model via anticholinergic receptors. In another study, Zhu et al. [50] reported that paeoniflorin extracts from *Radix Paeoniae Alba* inhibited cyclooxygenase, and that total glucosides from the paeony can play an anti-inflammatory role via the arachidonic acid metabolism. Pharmacological experiments have also demonstrated that *Ramulus Cinnamomi* exhibits antibacterial, anti-inflammatory, antiviral, antitumor, antipyretic, analgesic, antidiabetic, and antiplatelet aggregation effects. A previous study revealed that essential oil prepared from *Ramulus Cinnamomi* can exert anti-inflammatory effects by reducing the production of NO and PGE2 [51, 52].

## 5. Conclusions

This study provides evidence to support the protective mechanisms of GGD against PD. GGD exhibited significant analgesic effects in the treatment of PD by regulating oxytocin and estrogen signaling pathways in a rat model of PD. Our data suggest that the mechanisms underlying the protective effects of GGD on PD include the downregulation of HSP90AB1 and the reduced phosphorylation of three MAPKs (ERK1/2, p38, and JNK). Our data provide guidance and a useful foundation for investigating new treatments for PD.

## Figures and Tables

**Table 1:** Composition of Ge-Gen decoction.

Component	Part	Weight (g)
Pueraria lobata (Ge Gen)	Root	20
Ephedra (Ma Huang)	Twigs	5
Cinnamon twig (Gui Zhi)	Twigs	15
Paeonia lactiflora (Shao Yao)	Root	15
Glycyrrhizae radix et rhizome (ZhiGanCao)	Rhizome and root	10
Ginger (Sheng Jiang)	Root	10
Red dates (Hong Zao)	Fruit	20

## Data Availability

All of the data reported in this article are available from the corresponding author upon reasonable request.

## Abbreviations

GGD: Ge-Gen decoction
PD: Primary dysmenorrhea
DEPs: Differentially abundant proteins
PGE2: Prostaglandin E2
PGF2*α*: Pros*taglandi*n F2*α*
NSAIDs: Nonsteroidal anti-inflammatory drugs
GO: Gene Ontology
KEGG: Kyoto Encyclopedia of Genes and Genomes
LC-MS/MS: Liquid chromatographymass spectrometry/mass spectrometry
PMSF: Phenylmethane sulfonyl fluoride
PVDF: Polyvinylidene difluoride
PPI: Protein-protein interaction
HPLC-Q-TOF-MS/MS: High-performance liquid chromatography-quadrupole time-of-flight tandem mass spectrometry
SDS-PAGE: Sodium dodecyl sulfate-polyacrylamide gel electrophoresis
TBST: Tris buffered saline tween
RIPA: Radio immunoprecipitation assay
BCA: Bicinchoninic acid.
